# Protein Creatinine ratio versus 24-h test in HIV-positive and HIV-negative women with pre-eclampsia

**DOI:** 10.4102/safp.v68i1.6186

**Published:** 2026-04-24

**Authors:** Sinikeziwe F. Mkhize, Jagidesa Moodley, Olive P. Khaliq, Terrence Moodley

**Affiliations:** 1 Department of Obstetrics and Gynaecology, Faculty of Health Sciences, University of KwaZulu-Natal, Durban, South Africa; 2 Department of Paediatrics and Child Health, Faculty of Health Sciences, University of the Free State, Bloemfontein, South Africa

**Keywords:** protein/creatinine ratio, 24-hour test, pre-eclampsia, HIV, pregnancy

## Abstract

**Background:**

Pre-eclampsia (PE) is a major contributing factor to high rates of maternal and perinatal morbidity and mortality in South Africa. Early diagnosis is currently a challenge. The objective is to assess the diagnostic accuracy of spot urine protein-creatinine (P/C) ratio (UPCR) in comparison with 24-h urine proteinuria assessment for predicting PE.

**Methods:**

This was a prospective cross-sectional analytical study. A total of 125 women were included in the study and divided into normotensives (*n* = 25), early onset PE (*n* = 25), late onset PE (*n* = 25), chronic hypertension (*n* = 25) and gestational hypertension (*n* = 25) groups. Spot urine P/C ratio was determined in a mid-stream urine sample, and the 24-h urine protein was measured by standard laboratory techniques, and comparisons of the results were made between the groups.

**Results:**

A significant difference was found in the spot protein-creatinine ratios (PCRs) between normotensives and early onset PE (*p* < 0.0001) and between normotensives and late onset PE groups (*p* = 0.001). No significant differences were found between the 24-h urine and spot protein-creatinine across all groups: normotensives (*p* = 0.18); early onset PE (*p* = 0.63); late onset (*p* = 0.60); chronic hypertension (*p* = 0.43) and gestational hypertension (*p* = 0.95). In women with human immunodeficiency virus (HIV), no statistical significance was noted between the 24-hour test and the spot PCR.

**Conclusion:**

Spot PCR is a reliable test for quantifying proteinuria in all pregnant women, as the results were similar to that of a 24-h urine proteinuria test regardless of their HIV status. This shows that the spot PCR yields accurate results. The 24-hour test is inconvenient for pregnant women with and without HIV and is also expensive and time-consuming.

**Contribution:**

The UPCR can be introduced as an additional gold standard of PE analysis, especially in outpatients.

## Introduction

Hypertensive disorders of pregnancy (HDP), in particular, pre-eclampsia (PE) with severe features and eclampsia are life-threatening pregnancy conditions. These disorders affect 5% – 10% of all pregnancies and are substantial causes of maternal and perinatal morbidity and mortality globally.^1^ Hypertensive disorders of pregnancy are the commonest direct cause of maternal mortality and account for 14.8% of all maternal fatalities in South Africa. All types of pregnancy-related hypertension diseases can result in death, with eclampsia and PE being the most frequent final causes.^2^ Early detection and appropriate clinical management are required to mitigate against maternal morbidity and mortality.^3^ Hypertensive disorders of pregnancy are defined as new-onset hypertension (increase in blood pressure levels of ≥ 140/90 mmHg at 20 weeks or more of gestation).^4^ There are different subtypes of HDP, which are distinguished by their clinical characteristics, severity of the condition and the gestational age at onset (Table 1).^5^

**TABLE 1:** Hypertensive disorders of pregnancy.

Hypertensive disorder of pregnancy	Definition
Chronic hypertension	Occurs before pregnancy with a systolic blood pressure above or equal to 140 mmHg, and a diastolic blood pressure above or equal to 90 mmHg.^6^
Gestational hypertension	A blood pressure level that is equal to or above 140 mmHg/90 mmHg after 20 weeks of pregnancy.^6^
Pre-eclampsia	The occurrence of high blood pressure of 140 mmHg/90 mmHg with or without proteinuria after 20 weeks of pregnancy but with evidence of one or more thrombocytopenia, liver and kidney injury, severe epigastric pain, pulmonary oedema, persistent headaches is labelled pre-eclampsia.^7^
Eclampsia	Marked by the abrupt onset of seizures without any other neurological explanation.^8^
Chronic hypertension with superimposed pre-eclampsia	Women with primary or secondary chronic arterial hypertension who develop pre-eclampsia are referred to as having superimposed pre-eclampsia.^9^

Note: Please see the full reference list of the article Mkhize SF, Moodley J, Khaliq OP, Moodley T. Protein Creatinine ratio versus 24-h test in HIV-positive and HIV-negative women with pre-eclampsia. S Afr Fam Pract. 2026;68(1), a6186. https://doi.org/10.4102/safp.v68i1.6186

Obesity, a family history of hypertension, alcohol use, heart failure, stroke, left ventricular hypertrophy, and smoking are among the risk factors for hypertensive problems during pregnancy that have been found as risk factors associated with HDP.^10^ Adverse outcomes include placental hypoxia, resulting in intrauterine death, premature birth, small for gestational age foetuses, and high rates of maternal and perinatal morbidity and mortality.^11^ Because the exact cause of HDP remains unknown, the only known cure of HDP is delivery of the placenta, and this may require termination of the pregnancy prior to the onset of spontaneous labour.^12^ Furthermore, HDP complications may persist up to 12 weeks post-partum and in some cases result in long-term cardiovascular-metabolic effects, such as chronic hypertension and diabetes later in life.^13^

The early prediction of HDP in pregnant women is critical in the mitigation against high rates of maternal and neonatal mortalities as it alleviates morbidity and mortality associated with this pregnancy-specific condition. The current standardised method of diagnosis at antenatal clinics is measuring blood pressure and detecting protein in the urine of women at 20 weeks of gestation.^14^ Increasing levels of proteinuria are considered to be associated with higher rates of adverse maternal and foetal outcomes.^15^ Measurement of 24-h urine protein is considered to be a gold standard for the detection of proteinuria in the diagnosis of PE.^16^ The 24-h urine sampling, however, is time-consuming, inconvenient and cumbersome both for pregnant women and the staff supervising the urine collection.^17^ It is also subject to error because of inaccurate timing and/or incompleteness in the collection of the urine sample.^17^ Measuring the protein-creatinine ratio (PCR) in a spot urine sample is an alternative technique for quantitatively evaluating proteinuria. This method minimises the impact of fluctuations in urine concentration and offers a quicker and more convenient way to evaluate protein excretion.^18^ However, the correlations between spot urine samples and 24 h proteinuria remain controversial.^19^

Numerous studies have investigated the validity of the PCR test versus the 24-h urine test; however, results obtained by a number of researchers have revealed contradictory results because of a variation in the units used for urine protein and urine creatinine cut-off points – mg/mmol, mg/g, mg/mg, and g/g.^20^ Sensitivity and specificity for eight cut-off points were reported by nine studies, with a median of 24 mg/mmol urine protein-creatinine ratio (UPCR) (range 17 mg/mmol – 57 mg/mmol; 0.15 mg/mg – 0.50 mg/mg).^21^ However, the cut-off value for spot PCR was standardised in the early millennium (> 30 mg/mmol), and it was suggested that it could be used as an alternative to the 24-h urine test.^22^ Presently, spot urine PCR is used in some countries as a confirmatory test following screening by a standard urinary dipstick test. This is because both the visual dipstick and the automated dipstick showed low sensitivity (visual 56.0% and automated 53.8%).^23^ This test may also be useful in either confirming or ruling out proteinuria in patients with gestational hypertension.^23^ A comparison between the spot PCR and the 24-h urine testing was also performed in a study. The sensitivity and specificity of a random urine protein-to-creatinine ratio of ≥ 0.19 for the prediction of significant proteinuria were examined, and a receiver operating characteristic curve was created to ascertain the ideal cut-off value using the criterion of 24-h proteinuria of at least 300 mg as a significant proteinuria. A 100% sensitivity and 53.8% specificity were shown at a cutoff of 0.19. Significant proteinuria may be ruled out if the ratio is less than 0.22. With sensitivity, specificity, and accuracy of 96.6%, 92.3%, and 95.2%, respectively, the ideal cut-off value is 0.25.^24^

The current recommendation by the South African Society of Obstetrics and Gynaecology to measure proteinuria is as follows. Proteinuria above a trace (≥ +1), ≥ 0.3 g of proteinuria using a 24-h urine test, and a protein/creatinine ratio of ≥ 0.3 can be used to confirm pre-eclampsia.^25^ In South Africa (SA), the accuracy of spot urinary microalbumin : creatinine ratio and the visual dipstick test was performed in HDP. The results of this showed that neither the visual dipstick nor the microalbumin : creatinine ratio was accurate compared to the 24-h urine test. This was suspected to have been because of differences in total urinary protein in PE or technical issues with the assay that was used (Clinitek 50 systems).^26^

Both PE and human immunodeficiency virus (HIV) constitute common conditions in the South African population. In the province of KwaZulu-Natal, the prevalence of HIV infection among pregnant women is 40%, and HIV infection in PE is 12%.^27^ The frequency of PE is controversial in HIV-positive women. The rate of PE is reported to be lower in HIV-infected pregnancies,^28^ while other researchers reported that HIV-infected women have a significantly higher risk of PE development compared to HIV-uninfected women.^29^ However, the risk of PE increases with highly active antiretroviral therapy (HAART) administration, which induces immune reconstitution.^29^

Furthermore, HIV-positive pregnant women are potentially prone to having urinary tract infections (UTI), which may complicate the diagnosis or evaluation of proteinuria. Even though patients on HAART have increased chances of survival, their renal function is compromised over time.^30^ Therefore, HIV-infected women on treatment are predisposed to chronic kidney disease (CKD).^31^

Because PE can manifest without proteinuria but with new-onset hypertension and signs of involvement in one or more organ systems, including thrombocytopenia, liver and renal failure, and pulmonary oedema, proteinuria is currently not the primary predictor of PE.^7^ Moreover, proteinuria is one of the first signs of CKD in HIV-infected women. Previous studies have shown high rates of proteinuria in HIV-infected individuals in Africa^32^ and HIV-infected women in the USA.^33^ A study conducted by Jao et al. has reported that the use of combination antiretroviral therapy (cART) appears to be protective against proteinuria.^34^ Their findings were consistent with previous studies, which have shown cART to improve renal function in African HIV-infected individuals.^35^ Although the link between HAART use and PE development still remains elusive. More research is still needed on the association between HIV infection and PE development for early diagnostic purposes in order to reduce maternal and neonatal mortality.

There is a paucity of data in our setting in an HIV pre-eclamptic population comparing PCR in a spot urine sample against 24-h urine proteinuria. With the increasing incidence of HIV infection in our geographical region, the development of PE is increasing as well. In South Africa, 7.7 million people were living with HIV in 2023, with 4.9 million women of reproductive age (15–49 years).^36^ A study in South Africa reported that HIV-positive individuals develop PE upon receiving antiretroviral therapy.^37^ Therefore, it is important that rapid and evidence-based reliable methods of quantifying proteinuria in HIV-infected women be available. We therefore prospectively evaluated the accuracy of protein/creatinine ratio in the detection of significant proteinuria compared to 24-h urine protein excretion in HIV-infected women with PE.

## Research methods and design

This was a consecutive recruitment of pregnant women with and without HIV seen for antenatal care at a regional hospital in, KwaZulu-Natal, South Africa that covers primary, secondary and specialised care to pregnant women. This was a prospective cross-sectional analytical study design. The study was conducted from 2020 to 2021. All demographic information and urine samples were collected following consent from participants. All pregnant women ≥ 18 years with a gestational age > 20 weeks were included in the study. All women with known chronic renal diseases and chronic liver diseases and those ≤ 18 years were excluded from participation. Regulatory, ethical and health authority approvals for this study were obtained from the Biomedical Research Ethics Committee (BREC No: BE530/18) and the KwaZulu-Natal Department of Health. All research participants provided written informed consent. Participants’ demographic information such as age, parity, gravidity and clinical parameters such as blood pressure, proteinuria, and HIV status were recorded.

Women diagnosed to have PE based on a blood pressure level of ≥ 140/90 mm Hg and at least proteinuria ≥ 1^+^ by the standard dipstick method were included in the study. Based on the gestational age, women were classified as those having early- or late-onset PE. Early-onset PE (EOPE) was defined as that occurring prior to 33 weeks plus 6 days gestational age, while late-onset PE (LOPE) was defined as that occurring at 34 weeks gestational age and greater. Urine protein was assessed by the dipstick method, and the presence or absence of proteinuria was observed. If proteinuria was present, the grade, that is, trace, 1^+^, 2^+^, 3^+^, and 4^+^ was found, and proteinuria ≥ 1^+^ was considered as significant proteinuria. The amount of protein excretion was quantified in 24-h urine collected the next day, and the urine protein/creatinine ratio was calculated in a spot mid-stream urine sample. Protein levels of 0.3 g and above were regarded as positive, and any value below that was regarded as negative. The comparison between the accuracy of the spot protein/creatinine ratio and 24-h urine protein excretion was assessed.

### Data analysis

Results obtained in this study were analysed using GraphPad Prism, V 5.03 (Graph-Pad Software Inc, CA, United States). All parametric data were represented as mean ± s.d., while non-parametric data were presented as median and interquartile range. The Kruskal–Wallis test was performed for parametric tests to determine statistical significance between two or more independent variables. A paired t-test was performed to compare two groups, while the one-way ANOVA test was performed to compare more than two groups. In this study, a *p*-value of < 0.05 was considered significant.

### Sample size calculation

An institutional biostatistician determined the sample size. Cohen’s effect was used to calculate the sample size. To assess differences in mean proteinuria between study groups with an 80% power (1 beta [type 2 error probability]) and 95% confidence (or 5% alpha error probability [type 1]) using a student’s *t*-test, a sample size of 125 participants, 25 in each group, was required.

### Ethical considerations

Regulatory Ethical and Health Authority approvals for this study were obtained from the University of KwaZulu-Natal Biomedical Research Ethics Committee on 16 March 2022. The ethical clearance number is BREC/00002824/2021. All research participants provided written informed consent prior to participation.

## Results

The total sample population was 125. The study population was divided into four groups. Normotensives (*n* = 25), PE (*n* = 50), which was subdivided into early- (*n* = 25) and late-onset PE (*n* = 25), gestational hypertension (*n* = 25) and chronic hypertension (*n* = 25). Of the 125 women, 45 were HIV-positive (36%). The mean maternal age in the normotensive group was 27.40 ± 6.22, chronic hypertension (CH), 32.00 ± 6.45 and in gestational hypertension (GH), it was 30.30 ± 6.47. PE was divided into early-onset (EO) and late-onset (LO) PE, with the mean ages of 30.64 ± 6.63 and 30.28 ± 7.26, respectively. The mean gestational ages between the different groups were as follows: normotensives (31.96 ± 5.35), EO (26.92 ± 4.53), LO (35.36 ± 2.42), CH (34.24 ± 4.46), and GH (34.65 ± 4.44). The mean parity of normotensives was 1.38 ± 1.12; in EO, it was 1.50± 1.89, in LO, 1.27 ± 1.20, in CH, 1.35 ± 0.88, and in GH, 1.48 ± 1.11. The average proteinuria measured using the urinary dipstick in normotensives was 0.08 in EO, 1.10 in LO, 0.50 in CH, 0.32 and in GH, 0.18.

There were no significant differences in terms of demographics in maternal age normotensives versus early-onset, late-onset and gestational hypertension. A significant difference was found between normotensives and chronic hypertension (*p* = 0.03). No significant differences were found in gestational age between normotensives and chronic and gestational hypertension. However, significant differences were found between normotensives and early-onset PE, (*p* = 0.0002) and between normotensives and late-onset PE (*p* = 0.01). Statistically significant differences in systolic and diastolic blood pressures were found between normotensives and all HDP groups (*p* < 0.0001). No significant differences were found in proteinuria between normotensives and chronic hypertension and gestational hypertension. Moreover, proteinuria was statistically significant between normotensives and early-onset (*p* = 0.02) and late-onset PE (*p* = 0.02) (Table 2).

**TABLE 2:** Demographic and clinical variables of patient population (*N* = 25).

Characteristics	Normotensive	Early-onset PE	Late-onset PE	Chronic	Gestational hypertension	*p*
Maternal age (years)	27.40 ± 6.22	30.64 ± 6.63	30.28 ± 7.26	32.00 ± 6.45	30.30 ± 6.47	NT vs EO = 0.10 NT vs LO = 0.05 NT vs CH = *0.03 NT vs GH = 0.24
Gestational age (weeks)	31.96 ± 5.35	26.92 ± 4.53	35.36 ± 2.42	34.24 ± 4.46	34.65 ± 4.44	NT vs EO = ***0.0002 NT vs LO = *0.01 NT vs CH = 0.16 NT vs GH = 0.09
Gravidity	2.29 ± 1.16	2.96 ± 1.97	2.48 ± 1.36	2.48 ± 0.96	2.78 ± 1.63	NT vs EO = 0.34 NT vs LO = 0.60 NT vs CH = 0.71 NT vs GH = 0.89
Parity	1.38 ± 1.12	1.50± 1.89	1.27 ± 1.20	1.35 ± 0.88	1.48 ± 1.11	NT vs EO = 0.49 NT vs LO = 0.83 NT vs CH = 0.91 NT vs GH = 0.34
Systolic (mmHg)	110.50 ± 14.29	155.80 ± 19.78	146.96 ± 9.24	142.88 ± 22.68	150.65 ± 9.33	NT vs EO = ***< 0.0001 NT vs LO = ***< 0.0001 NT vs CH = ***< 0.0001 NT vs GH = ***< 0.0001
Diastolic (mmHg)	65.50 ± 13.32	99.40 ± 10.01	94.36 ± 7.19	89.96 ± 17.10	88.13 ± 10.59	NT vs EO = ***< 0.0001 NT vs LO = ***< 0.0001 NT vs CH = ***< 0.0001 NT vs GH = ***< 0.0001
Proteinuria	0.08	1.10	0.50	0.32	0.18	NT vs EO = *0.02 NT vs LO = *0.02 NT vs CH = 0.22 NT vs GH = 0.64

PE, pre-eclampsia; NT, normotensive; EO, early-onset; LO, late-onset; CH, chronic; GH, gestational hypertension.

* , indicates that a result is statistically significant with a *p*-value less than 0.05 (*p* < 0.05);

*** , indicates a highly significant result, where the *P*-value is less than 0.001 (*p* < 0.001).

**FIGURE 1:**
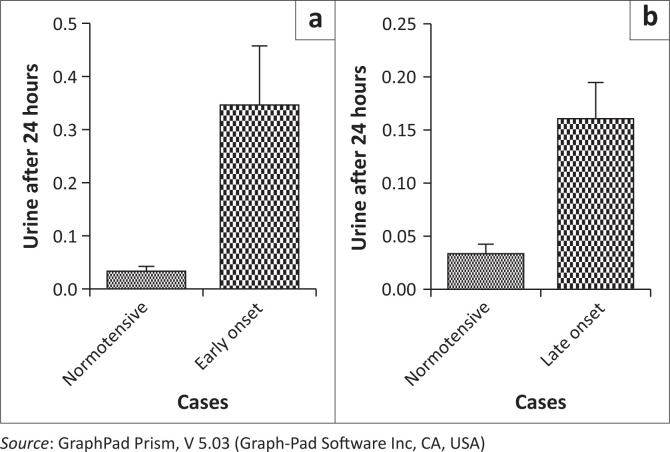
Comparison of the 24-hour urine test in (a) normotensive early onset cases versus (b) normotensive late onset cases.

**FIGURE 2:**
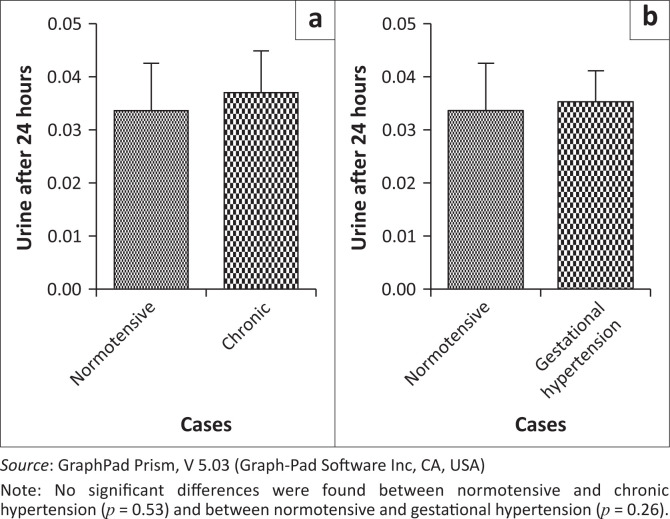
The 24 hour urine test between (a) normotensive and chronic hypertension cases versus (b) normotensive and gestational hypertension.

The 24-h urine analysis was performed in all groups. The 24-h urine analysis was statistically significant between normotensives and both EOPE and LOPE (*p* < 0.0001) (Figure 1). However, no statistical differences were found between normotensives and chronic hypertension (*p* = 0.53) and between normotensives and gestational hypertension (*p* = 0.26) (Figure 2).

**FIGURE 3:**
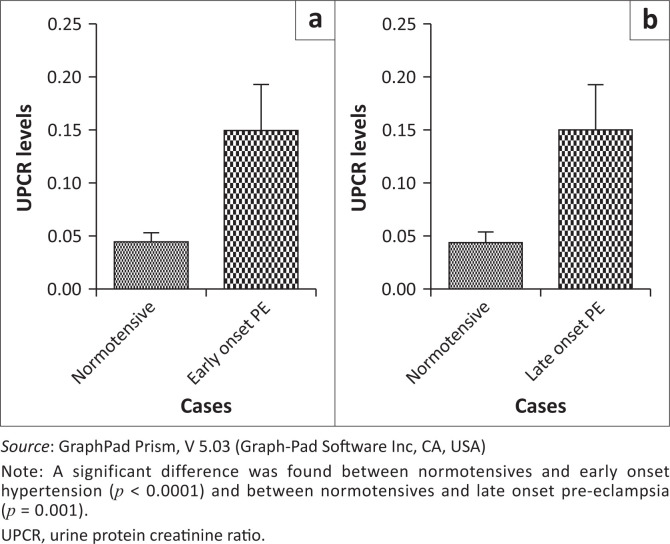
Urine protein-creatinine ratio comparison between (a) normotensive and early onset pre-eclampsia, and (b) normatensive and late onset pre-eclampsia.

**FIGURE 4:**
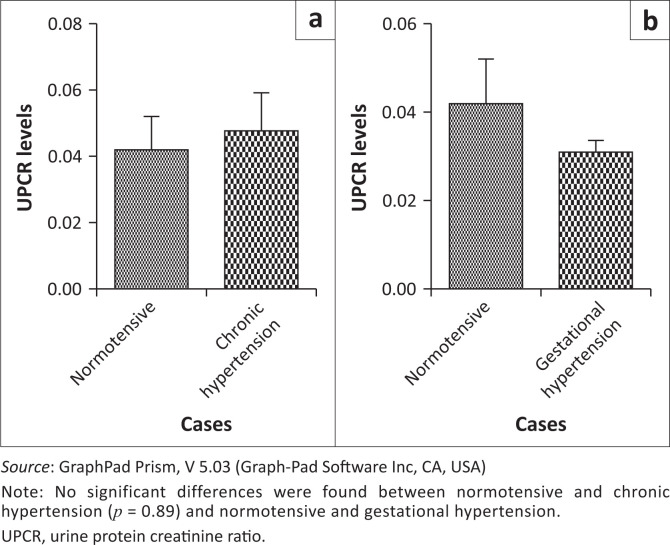
Urine protein-creatinine ratio comparison between (a) normotensive and chronic hypertension and (b) normotensive and gestational hypertension.

**FIGURE 5:**
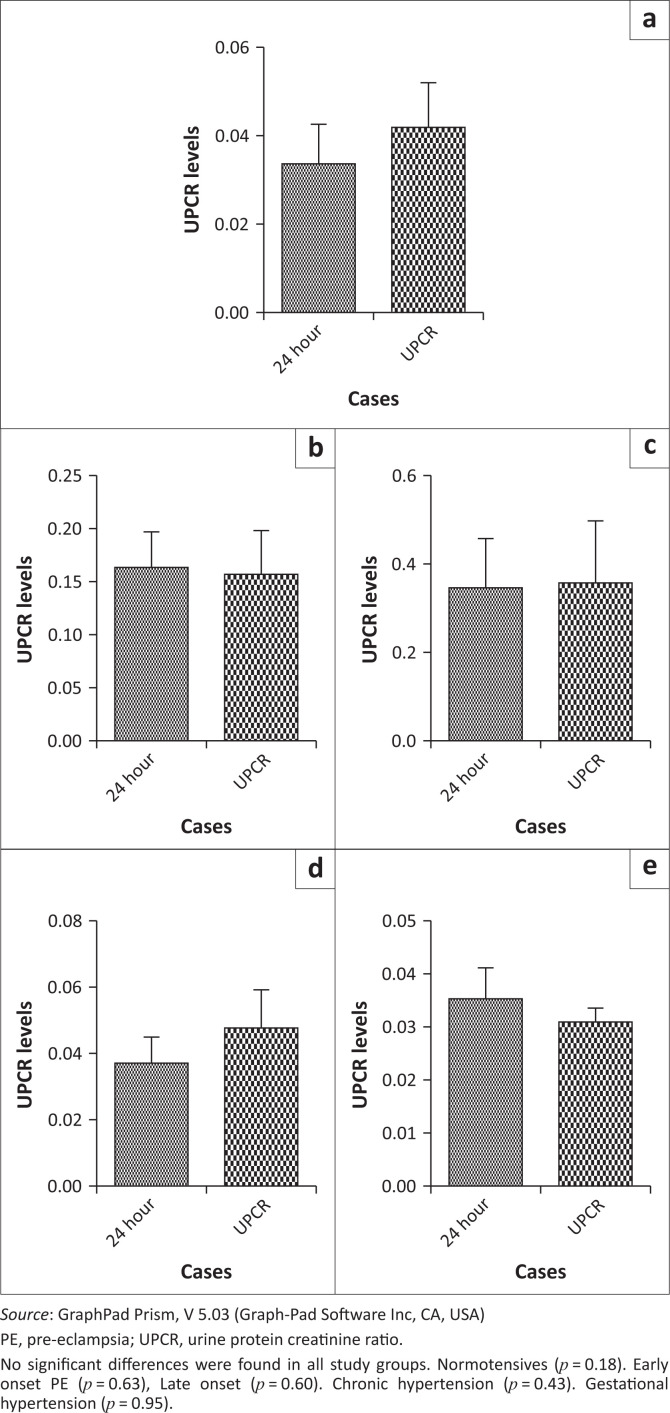
The comparison between the 24-h urine test and the protein-creatine ratio in (a) normotensive cases; (b) late onset pre-eclampsia cases; (c) early onset pre-eclampsia cases; (d) chronic hypertension cases; and (3) gestational hypertension cases.

A significant difference was also found in spot PCRs between normotensives and EOPE (*p* < 0.0001) and between normotensives and LOPE (*p* = 0.001) (Figure 3). No significant differences were found in spot protein-creatine ratios between normotensives and chronic hypertension (*p* = 0.89) and between normotensives and gestational hypertension (*p* = 0.51) (Figure 4). A comparison was carried out between the 24-h urine test and the spot PCR in all groups. No significant differences were found between the results of the two tests in all groups. There was no statistically significant difference between the 24-h urine test and the spot PCR in normotensives (*p* = 0.18), EOPE (*p* = 0.63), LOPE (*p* = 0.60), chronic hypertension (*p* = 0.43) and gestational hypertension (*p* = 0.95) (Figure 5).

**FIGURE 6:**
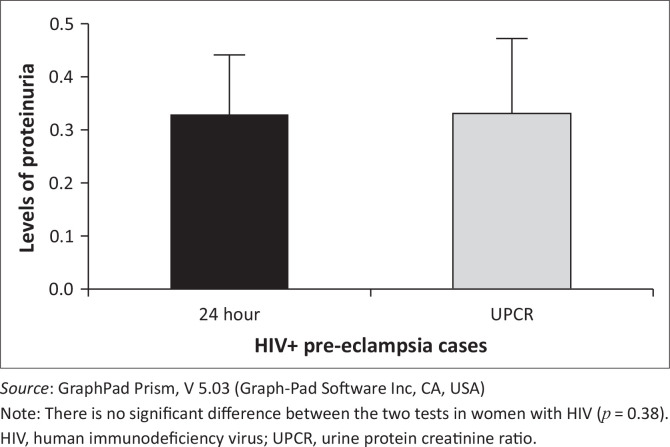
The comparison between the 24-hour urine test and the spot UPCR test in HIV+ pre-eclamptic women.

**FIGURE 7:**
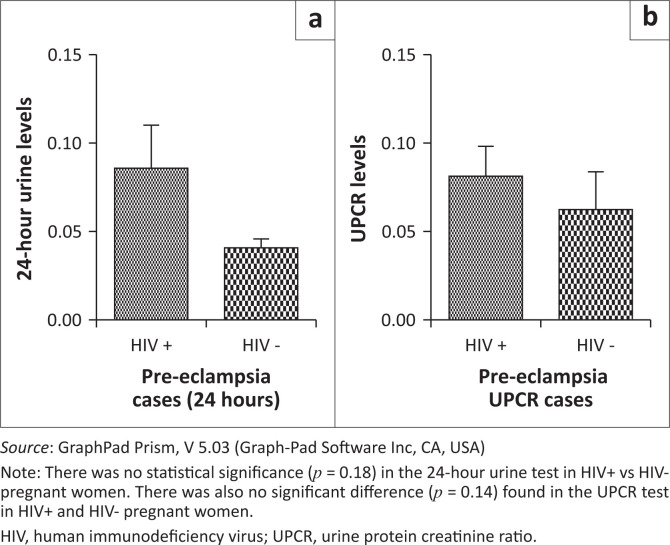
Comparison of (a) 24-hour urine test and (b) the spot UPCR in HIV+ and HIV- women with pre-eclampsia.

A comparison between the 24-h urine test and urine protein creatinine ratio in HIV pre-eclamptic women showed no significant difference (*p* = 0.38). (Figure 6). There was also no statistical significance (*p* = 0.18) in the 24-h urine test in HIV positive and HIV negative pregnant women. No significant difference (*p* = 0.14) was found in the urine protein creatinine ratio test in HIV positive and HIV negative pregnant women (Figure 7).

## Discussion

This study found no significant differences between the 24-h urine test and the spot protein-creatinine ratio across all study groups with and without HIV infection. Thus, HIV may not have an impact on proteinuria results in a 24-h urine test versus a spot PCR in pregnant women with HPD. These findings are in line with a study conducted in the USA, which found that the spot PCR and the 24-h urine test results were the same in women with PE (there was an 81.1% positive agreement between the two tests, and 71.4% negative agreement).^38^ Furthermore, another study performed in Turkey reported similar results and concluded that the spot PCR can be used as a predictor for proteinuria in women suspected to have PE.^39^

Lamontagne et al. reported that the spot protein-creatinine is a reliable test for the detection of proteinuria, but that caution should be taken in terms of the timing of the test. Their study showed that the accuracy of the tests is reduced in samples taken for the first time in the morning.^40^ Contrary to our findings, a Turkish study reported that the spot PCR was a poor detector of proteinuria compared to the 24-h urine test.^41^ Another study that contradicts this study’s findings was performed in India, which found that a random spot PCR was a poor predictor of proteinuria in women with PE.^42^

Recently, a similar study to the current one was conducted in the Free State Province, South Africa, and showed that the spot PCR is a reliable and accurate predictor of proteinuria in women with pre-eclampsia and can be used instead of the 24-h urine test because it is less time-consuming and cost-effective.^43^ The Free State study found a significant difference between normotensive women and women with early-onset pre-eclampsia in the 24-h urine test (*p* < 0.0001) and the spot protein-creatine ratio (*p* < 0.0001). In addition, a significant difference was found between normotensive women and women with late-onset pre-eclampsia in the 24-h urine test (*p* < 0.0001) and the spot PCR (*p* = 0.001).^28^ This shows that both tests can accurately detect proteinuria in women with PE. However, a study conducted in Taiwan reported contradictory results and found no statistical significance in proteinuria in normotensive women compared to women with pregnancy-induced hypertension using the spot PCR.^44^

Proteinuria is currently not the main predictor of PE because this disorder has been recognised to occur without proteinuria but with new-onset hypertension and evidence of one or more organ system involvement such as such as thrombocytopenia, liver and renal dysfunction and pulmonary oedema.^7^ However, proteinuria is critical in distinguishing among the various subtypes of HDP. The diagnosis of gestational hypertension is made by the exclusion of proteinuria, and this HDP subtype was included in this study. There were no significant differences between the 24-h urine test and the spot PCR in the gestational hypertensive groups. There was also no significant difference between the normotensive group proteinuria and the gestational hypertensive group proteinuria using the 24-h urine test (*p* = 0.26) and the spot PCR (*p* = 0.89). This indicates that the spot PCR is a reliable test to predict negative proteinuria results as well.

### Study limitations

There was an unequal distribution of HIV-positive and HIV-negative women in the different groups included. This made it difficult to compare the levels of proteinuria in HIV-positive women compared to HIV-negative women. In addition, the sample size was small compared to other studies in the literature. The findings of this study need to be confirmed by other studies with a larger sample size, with an equal distribution between the HIV-positive and HIV-negative groups. Furthermore, a validity test is required to ascertain the use of UPCR as a gold standard.

### Recommendations

A larger sample size is required with equal numbers of HIV-positive and HIV-negative women to predict the accuracy of the spot PCR. It would also be beneficial to use the spot protein-creatinine ratio instead of the 24-h urine test in all pregnant women suspected to be at risk of developing HDP because the test is convenient and less time-consuming, whether performed on an outpatient or inpatient basis.

## Conclusion

This study found the spot PCR to be a good test for the detection of proteinuria in women with HDP and that this test can be used as an alternative test to the 24-h urine test, which is inconvenient for women, expensive and time-consuming compared to the spot protein-creatinine test. The study also showed that there was no significant difference between the 24-h urine test and the spot protein-creatinine ratio in pre-eclamptic women with HIV, an important aspect in countries in sub-Saharan Africa with high rates of both HIV and HPD. This study added to the body of knowledge in terms of proteinuria analysis in women with PE. The UPCR can be introduced as an additional gold standard of PE analysis, especially in outpatients.
